# Enhanced Electrical and Thermal Conductivities of Polymer Composites with a Segregated Network of Graphene Nanoplatelets

**DOI:** 10.3390/ma16155329

**Published:** 2023-07-29

**Authors:** Ki Hoon Kim, Ji-Un Jang, Gyun Young Yoo, Seong Hun Kim, Myung Jun Oh, Seong Yun Kim

**Affiliations:** 1 Department of Carbon Composites Convergence Materials Engineering, Jeonbuk National University, 567 Baekje-daero, Deokjin-gu, Jeonju-si 54896, Jeonbuk, Republic of Korea; kihoon2376@jbnu.ac.kr; 2 Research Institute of Industrial Science, Hanyang University, 222 Wangsimni-ro, Haengdang-dong, Seongdong-gu, Seoul 04763, Republic of Korea; jju204@hanyang.ac.kr (J.-U.J.); kimsh@hanyang.ac.kr (S.H.K.); 3 Department of Organic Materials and Textile Engineering, Jeonbuk National University, 567 Baekje-daero, Deokjin-gu, Jeonju-si 54896, Jeonbuk, Republic of Korea; ky5932@gmail.com

**Keywords:** composites, segregated network, electrical conductivity, thermal conductivity, graphene

## Abstract

Introducing a segregated network constructed through the selective localization of small amounts of fillers can be a solution to overcome the limitations of the practical use of graphene-based conductive composites due to the high cost of fillers. In this study, polypropylene composites filled with randomly dispersed GNPs and a segregated GNP network were prepared, and their conductive properties were investigated according to the formation of the segregated structure. Due to the GNP clusters induced by the segregated structure, the electrical percolation threshold was 2.9 wt% lower than that of the composite incorporating randomly dispersed GNPs. The fully interconnected GNP cluster network inside the composite contributed to achieving the thermal conductivity of 4.05 W/m∙K at 10 wt% filler content. Therefore, the introduction of a segregated filler network was suitable to simultaneously achieve excellent electrical and thermal conductivities at a low content of GNPs.

## 1. Introduction

Graphene is well known for its excellent conductive properties, such as charge mobility (~200,000 cm^2^/V·s [1,2]), electrical conductivity (~10^5^ S/m [3]), and thermal conductivity (3000−6500 W/m∙K [4,5]). In particular, graphene nanoplatelet (GNP)-filled conductive polymer composites (CPCs), which are lightweight, easy to process, and have excellent portability, are receiving a lot of attention because the GNPs manufactured via the top-down method of exfoliating graphite are advantageous in terms of price and mass production compared to bottom-up fabrication based graphene [6,7,8,9,10]. To maximize the electrical and thermal conductivities of CPCs, uniform filler dispersion has been identified as an important structural factor [11,12,13,14]. To achieve the uniform filler dispersion, various methods [15,16,17,18,19], such as covalent functionalization [15], noncovalent functionalization [16,18,19], and polymer wrapping [17], have been reported. However, despite these efforts, there is a need for a method for spreading the application of GNP-based CPCs by innovatively reducing the amounts of expensive GNPs incorporated into the composites.

Various strategies [20,21,22,23,24,25,26] have been proposed to achieve excellent electrical and thermal conductivities of polymer composites by incorporating smaller amounts of fillers. Double percolation can be generated by the selective localization of nanofillers based on thermodynamic (chemical affinity) and kinetic (melting point difference) factors between nanofillers and an immiscible matrix [20]. This strategy induces a predominant distribution of conductive fillers in one matrix phase. Hence, the amount of filler used to implement double-percolated CPCs with a certain conductive performance level is significantly smaller than that to implement randomly dispersed CPCs (R-CPCs) [21,22,23]. To further reduce the amount of conductive filler used, a segregated structure in which the filler is selectively localized at the interface has been proposed [24]. The segregated structure can minimize the proportions of fillers by forming a matrix region inside the network where fillers are not mixed [25,26,27,28,29]. The introduction of the segregated network can innovatively enhance the electrical and thermal conductivities of CPCs simultaneously. Therefore, there is a need to understand the simultaneous enhancement of electrical and thermal conductivities with respect to the structural development of GNP-based segregated composites.

In this study, the electrical and thermal conductivities of composites according to their network structures of conductive fillers were investigated experimentally and theoretically. GNP-based R-CPCs and segregated CPCs (S-CPCs) were prepared, and the electrical and thermal conductivities of the CPCs were analyzed. The percolation threshold in the electrical conductivity of the R-CPCs was 3 wt%, and the thermal conductivity of the R-CPC increased linearly according to the fitting of Nan’s model. In contrast, the electrical percolation threshold of the S-CPC was observed at 0.1 wt% (0.04 vol%), and the thermal percolation behavior where the thermal conductivities of the S-CPCs rapidly increased following the thermal percolation model was confirmed.

## 2. Materials and Methods

### 2.1. Materials

GNPs (M25, XG Science, Lansing, MI, USA) with a lateral size of 25 μm, a thickness of 5 nm, and a density of 2.2 g/cm^3^ [30], respectively, were used as fillers to improve the conductivities of the composites. Pelletized polypropylene (PP, Y-120A, Lotte Chemical, Daejeon, Republic of Korea), with an average diameter of 2–4 mm, was used as polymer matrix. The used PP exhibited a density of 0.9 g/cm^3^ and a melting temperature (T_m_) of 165 °C.

### 2.2. Composite Fabrication

**Figure 1:**
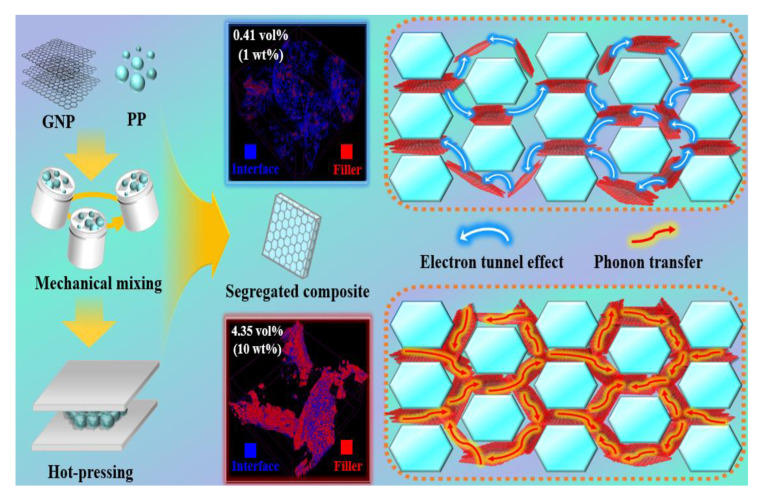
Schematic for fabrication process of S-CPC.

Fabrication process of S-CPC is shown schematically in Figure 1. Before fabricating the composite, the raw materials were dried overnight at 85 °C to remove moisture. PP and GNP were weighed at the target content and mixed at 2000 rpm for 2 min using a mechanical mixer (ARE 310, Thinky Corp., Tokyo, Japan). The mixture was hot-compacted using a heating press (D3P-20J, Dae Heung Science, Incheon, Republic of Korea) at 15 MPa and 160 °C (below T_m_) for 15 min. Fabrication of the segregated composite below T_m_ can lead to stable segregated networks that were fillers localized on the matrix interface and induces relatively superior conductive properties [26]. The composites with randomly dispersed GNP were fabricated by molding to size of 25 × 25 × 2 mm^3^ using hot pressing after stirring (60 rpm) at the temperature (180 °C) that the matrix melted completely (Figure S1). The segregated composite and the composite with randomly dispersed GNPs were labeled S-CPCX and R-CPCX, respectively. X implied the weight fraction of GNP. The compositions of the prepared composites are presented in Table 1.

### 2.3. Characterization

The morphologies of the composites were observed via a field emission electron microscope (FE-SEM, GeminiSEM 500, Zeiss, Oberkrochen, Germany). Before observation, the specimens were dipped in liquid nitrogen for 5 min and mechanically fractured. The surfaces of specimens were Pt-coated for 140 sec in vacuum via a sputtering machine (Ion Sputter E-1030, Hitachi High Technologies Co., Tokyo, Japan). The Pt-coated specimens were observed under a nitrogen condition with an applied voltage of 2 kV. Nondestructive three-dimensional (3D) analysis was conducted with micro-computed tomography (μ-CT, Skyscan 1172, Bruker Co., Billerica, MA, USA) to analyze the 3D internal structure of the specimen. The 3D image of the specimen was attained via X-ray irradiation and reconstructed using a software program (NRecon, Version 1.6.10.2). The high electrical resistance of the composite was measured via an ultrahigh resistance meter (SM-8220, HIOKI E. E. Corporation, Nagano, Japan). The low electrical resistance of the composite was measured based on direct current resistance (DC) using a Keithley 2400 Source Meter. Before the measurement, the specimens were coated with silver paste to reduce the contact resistance levels between the specimens and electrodes. The electrical conductivity (σ) of the specimen was calculated via the following equation:

(1) $$\sigma =\frac{L}{Rwt}$$

where R, L, w, and t are the measured electrical resistance, length, width, and thickness of the specimen, respectively. The isotropic thermal conductivity of the sample with 25 × 25 × 2 mm^3^ was analyzed using a hot-disk method (TPS 2500S, Hot disk AB, Gothenburg, Sweden) according to ISO 22007-2 [31].

### 2.4. Electrical Percolation Model

The theoretical electrical conductivities of the fabricated specimens are calculated via the electrical percolation equation [32]. The electrical conductivity of the composite ($\sigma_{c}$) is enhanced due to the electron tunnel effect induced by conductive particles (GNP fillers) located in the insulating matrix (PP). Prior to the filler content where the tunnel effect is maximized ($\phi_{c}$, percolation threshold), the electrical conductivity of the composite is expressed with the electrical conductivity of the matrix ($\sigma_{m}$) and the exponent (s). In addition, the $\sigma_{c}$ after $\phi_{c}$ is expressed with the electrical conductivity of the filler ($\sigma_{f}$) and another exponent (v). The critical percolation exponents—s and v—are governed by the size and shape of the conductive particle and the thickness of the insulating layer (=distance between GNP fillers), respectively. The slope reaching the saturation region of electrical conductivity is determined. In this study, the electrical conductivities of the segregated composites ($\sigma_{sc}$) are expressed as follows:

(2) $$\begin{array}{c} \sigma_{sc}=\sigma_{m}{\left[\left(\phi_{ec}\right)/\left(\phi_{ec}-\phi_{f}\right)\right]}^{s}\;\left(if,\;\phi_{f}<\phi_{ec}\right)\\ \sigma_{sc}=\sigma_{f}{\left[\left(\phi_{f}-\phi_{ec}\right)/\left(1-\phi_{ec}\right)\right]}^{v}\;\left(if,\;\phi_{f}>\phi_{ec}\right) \end{array}$$

where $\sigma_{m}$, $\phi_{ec}$, $\phi_{f}$, and $\sigma_{f}$ denote the electrical conductivity of the PP matrix (1.04 × 10^−13^ S/m), electrical percolation threshold of the segregated composite (0.04 vol%), GNP filler content and electrical conductivity of the GNP filler (10^4^ S/m [33]), respectively. In addition, s and v indicate the percolation exponents before and after the critical volume fraction, respectively.

### 2.5. Nan’s Model and Thermal Percolation Model

The thermal conductivity (${TC}_{c}$) of the composite incorporating the nanofiller is lower than expected for the rule of mixtures due to the interfacial thermal resistance (ITR, ≈ Kapitza radius) generated at the interface between the nanocarbon filler and the polymer matrix. Nan’s model is an effective tool for evaluating the thermal conductivity of composite because ${TC}_{c}$ can be predicted according to the size, shape, and content of the nanocarbon filler by assuming the uniform dispersion of fillers [34]. The theoretical thermal conductivities of the R-CPC and S-CPC fabricated in this study are described using Nan’s model as follows:

(3) $${TC}_{Nan}={TC}_{m}\times \left(\frac{3+\phi_{f}\times (\beta_{x}+\beta_{z})}{3-\phi_{f}\times \beta_{x}}\right)$$

where,

(4) $$\beta_{x}=\frac{2(K_{11}^{C}-{TC}_{m})}{K_{11}^{C}+{TC}_{m}},\beta_{z}=\frac{K_{33}^{C}}{{TC}_{m}}\;–\;1$$

${TC}_{m}$ is the thermal conductivity of the PP (0.30 W/m·K), $\phi_{f}$ is the volume fraction of GNP fillers, and $K_{11}^{C}$ and $K_{33}^{C}$ are the equivalent thermal conductivities of GNPs surrounded with parallel and perpendicular interfacial barrier layers of the unit cell, respectively, which can be described as following equation:

(5) $$K_{11}^{C}=\frac{{TC}_{f}}{1+\frac{{2a}_{k}{TC}_{f}}{h{TC}_{m}}},\;K_{33}^{C}=\frac{{TC}_{f}}{1+\frac{{2a}_{k}{TC}_{f}}{d{TC}_{m}}},\;a_{k}=R_{ITR}\times {TC}_{m}$$

where ${TC}_{f}$, $a_{k}$, and $R_{ITR}$ are the thermal conductivity of the GNP (3000 W/m·K), Kapitza radius (25.1 nm), and ITR (8.40 × 10^−8^ m^2^ K/W [35]), respectively, and h and d are the thickness (5 nm) and lateral size (25 μm) of the GNP, respectively.

(6) $${TC}_{P}={TC}_{m}\times \left(1-\phi_{f}\right)+{TC}_{o}{\left(\frac{\phi_{f}-\phi_{tc}}{1-\phi_{tc}}\right)}^{z}$$

where ${TC}_{P}$, ${TC}_{m}$, and $\phi_{f}$ are the theoretical conductivity of a composite filled with a thermally percolated filler network, the thermal conductivity of the matrix (0.30 W/m·K), and the volume fraction of GNP fillers, respectively. In this study, $\phi_{tc}$ is 0.0045 (0.45 vol%). ${TC}_{o}$ and z are the pre-exponential factor (91 W/m·K in this work) and critical exponent (0.98 in this work).

### 2.6. Applications

The improved electrical and thermal conductivities of the manufactured composites were applied as humidity sensors and thermal interface management (TIM) materials, respectively. After the prepared composite was placed on an electrode in an acrylic chamber connected to an external multimeter (Fluke 17B+ MAX Digital Multimeter, Fluke Corporation, Everett, WA, USA), surface resistance was measured according to humidity control (30−80% relative humidity (RH)) using a humidifier. Humidity-sensing sensitivity (HS) was calculated based on the measured resistance and humidity as following equation:

(7) $$HS\;\left(\%\right)=\frac{∆R}{R_{30}}\times 100$$

where, $R_{30}$ is the resistance at 30% RH and ∆R represents the difference between the resistances at 80% RH and $R_{30}$. In addition, the thermal images for the application of the TIM material were obtained by measuring the average surface temperature of the fabricated composite at 10 sec using a thermal camera (Testo 875 infrared thermal imager, Testo Ltd., Lenzkirch, Germany) after being placed on the hot plate at 100 °C.

## 3. Results and Discussion

**Figure 2:**
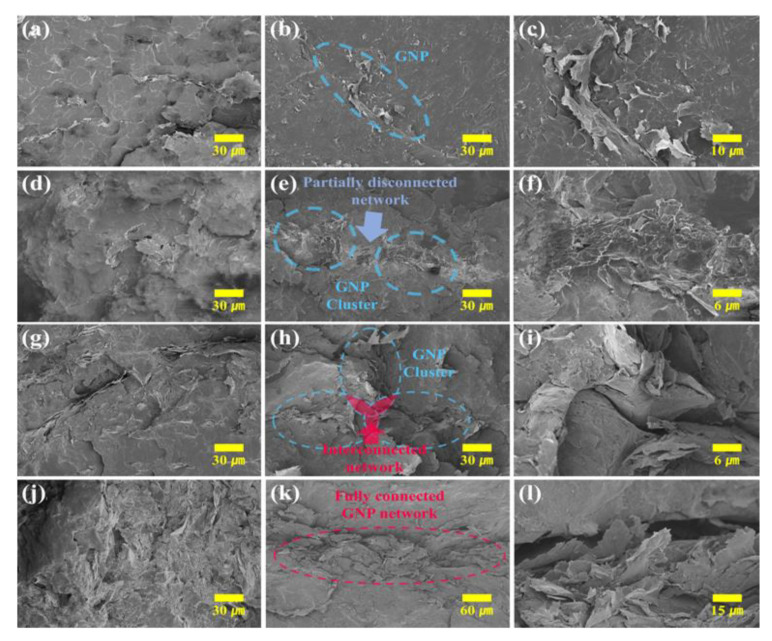
FE-SEM images of (**a**) R-CPC0.3 and S-CPC0.3 at (**b**) low and (**c**) high magnifications, (**d**) R-CPC1 and S-CPC1 at (**e**) low and (**f**) high magnifications, (**g**) R-CPC2 and S-CPC2 at (**h**) low and (**i**) high magnifications, and (**j**) R-CPC10 and S-CPC10 at (**k**) low and (**l**) high magnifications.

Cross-sectional FE-SEM images of the prepared specimens are placed in Figure 2a–l. A uniform dispersion of GNPs was confirmed in R-CPC0.3 (Figure 2a). In contrast, the selective localization of GNP in the interface of the segregated composite was confirmed in S-CPC0.3 (Figure 2b). In the magnified image of S-CPC0.3 (Figure 2c), GNPs were compacted at the interface, indicating that the segregated structure was formed in the S-CPC even at low filler contents. R-CPC1 and S-CPC1 showed more obvious differences in filler distribution (Figure 2d–f). A uniform GNP dispersion of R-CPC was observed, while S-CPC obviously exhibited a segregated structure. In addition, R-CPC showed an insulating gap (PP matrix) between GNPs due to uniform dispersion, and GNP clusters formed by the contact of incorporated fillers were observed in S-CPC. This morphological difference could affect the conductivities of the R-CPC and S-CPC. Despite the successful formation of GNP clusters in S-CPC, the partial disconnection between adjacent GNP clusters observed at the interface of the segregated structure indicated that an interconnected network was not formed at the 1 wt% filler content.

R-CPC2 still exhibited an insulating gap between the GNPs (Figure 2g). A few interconnected networks in which GNP clusters contacted each other were observed in S-CPC2 (Figure 2h,i). The presence of interconnected networks within the composite can greatly enhance tunneling conductivity and reduce phonon scattering, resulting in improved phonon transfer [36,37]. This indicates that fabricating segregated structures is an efficient strategy for forming a compact conductive network via selective localization of fillers. A uniform GNP dispersion of R-CPC10 was observed despite the maximum filler loading (Figure 2j). The reduced distances between fillers due to the high content could improve the electron tunnel effect and effective phonon transfer. From the observation of GNPs well localized at the matrix interface in S-CPC10, the applied process was suitable for fabricating segregated composites up to 10 wt% GNP loading (Figure 2k,l). In addition, a fully connected network (Figure 2k) was formed by GNP clusters consisting of fillers in contact (Figure 2l) with each other in the segregated composite. The fully connected filler network could contribute to the dramatic enhancements in conductivities.

**Figure 3:**
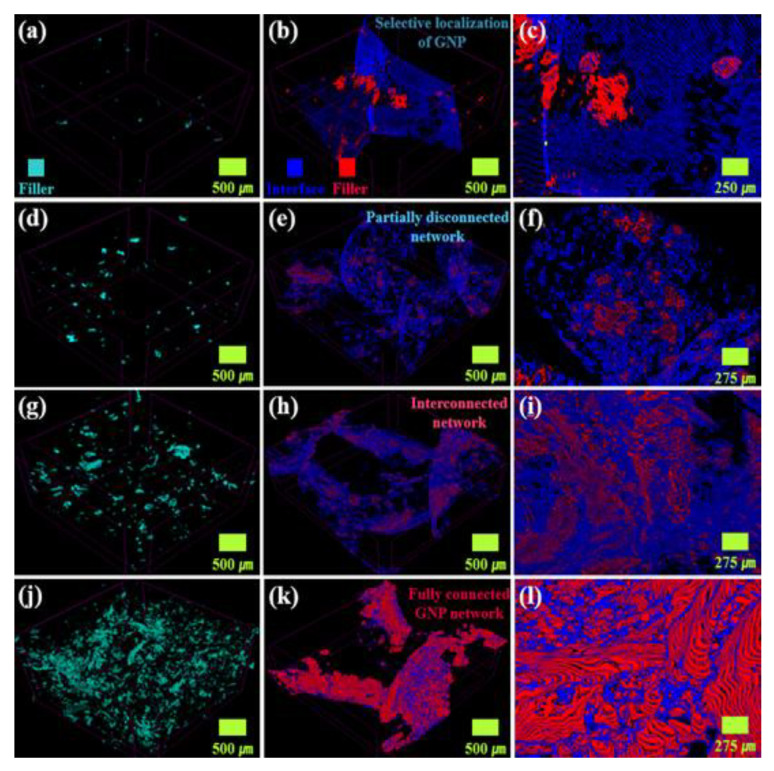
μ-CT images of (**a**) R-CPC0.3 and S-CPC0.3 at (**b**) low and (**c**) high magnifications, (**d**) R-CPC1 and S-CPC1 at (**e**) low and (**f**) high magnifications, (**g**) R-CPC2 and S-CPC2 at (**h**) low and (**i**) high magnifications and (**j**) R-CPC10 and S-CPC10 at (**k**) low and (**l**) high magnifications.

Nondestructive observation of the 3D segregated structure using μ-CT was performed. Figure 3a–c shows the μ-CT images of R-CPC0.3 and S-CPC0.3. In Figure 3a, uniformly dispersed GNPs within the PP matrix were observed. The obvious distances between the fillers as insulating gaps could result in the low conductivities of the composite. On the other hand, GNPs were selectively located at the matrix interface in S-CPC0.3 (Figure 3b,c) and formed a conductive pathway (Figure 3c). Clusters of GNPs were formed with the selective localization in S-CPC1 (Figure 3e,f). In addition, partial disconnection between adjacent GNP clusters was observed, as discussed in Figure 2e. In the case of S-CPC2, multiple GNP clusters in the segregated structure were observed (Figure 3h,i). The interconnections between the clusters identified in the high-magnification image could be beneficial for both electron tunneling and phonon transfer. Figure 3j–l shows the μ-CT images of the R-CPC10 and S-CPC10. Despite the high filler content, a uniform dispersion and segregated structures were induced. In particular, a fully connected network of S-CPC10 was clearly confirmed via nondestructive 3D structural analysis based on μ-CT and 2D observation using FE-SEM.

**Figure 4:**
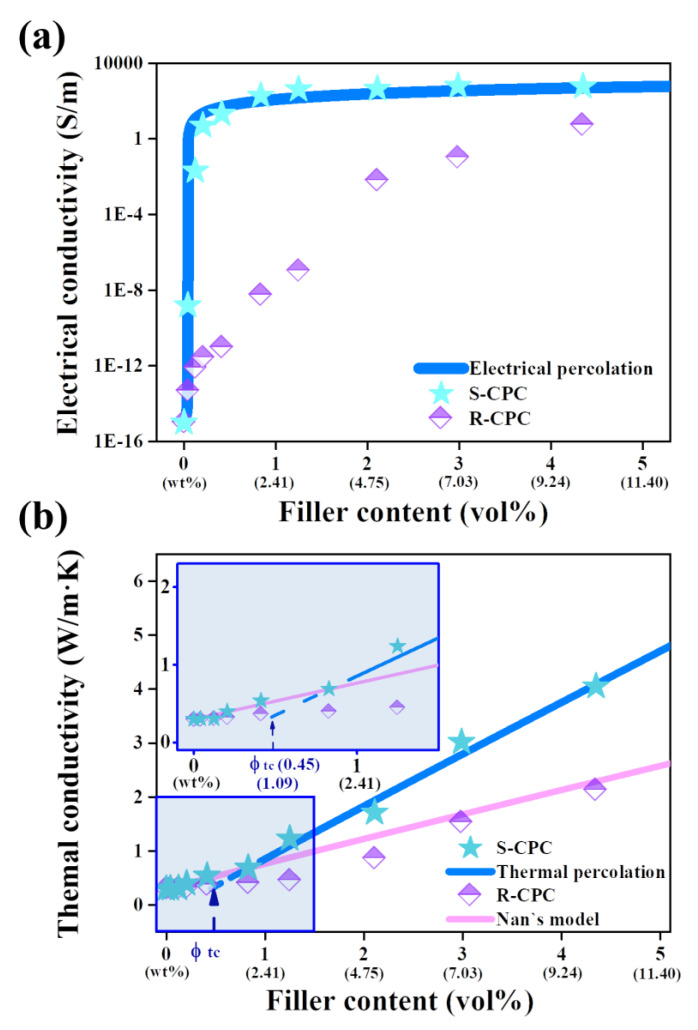
Experimentally and theoretically obtained (**a**) electrical and (**b**) thermal conductivities of the fabricated specimens.

The electrical conductivity of composite could be described by percolation theory based on the tunnel effects of electrons [38,39]. Electrical percolation behavior is represented by a dramatic improvement in the electrical conductivities of composites when fillers are located at a specific distance where the tunnel effect is generated [40]. The segregated structure could form a compact conductive network derived from the selective localization of fillers, achieving superior electrical conductivities of S-CPCs at low filler contents relative to R-CPCs [41,42]. A significant difference in the electrical conductivities of the R-CPC and S-CPC was observed in Figure 4a. For example, R-CPC0.3 and S-CPC0.3 achieved 7.90 × 10^−13^ S/m and 2.06 × 10^−2^ S/m, respectively, indicating that the electron tunnel effect was generated by the selective localization of the fillers based on the segregated structure. In addition, R-CPCs showed a moderate increase of the electrical conductivity up to 10 wt% (~5.81 S/m), whereas the electrical conductivity of S-CPC1 was 20.79 S/m, where GNP clusters were identified. Furthermore, the electrical conductivity of the S-CPC2 showed 192.87 S/m before saturation, where interconnections between adjacent GNP clusters were confirmed. Therefore, based on the internal structure analysis and electrical conductivity results, the segregated structure of the fabricated composite was effective for increasing the electrical conductivities and reducing the filler contents required for generating the electron tunnel effect. The theoretically evaluated electrical conductivity using the electrical percolation equation showed that the segregated composites were advantageous in inducing a reduced percolation threshold ($\phi_{ec}$ at 0.04 vol% (0.1 wt%)) compared to that of R-CPCs ($\phi_{ec}$ at 1.25 vol% (3 wt%)). Comparisons for the electrical percolation thresholds and maximum electrical conductivities of previously reported GNP-segregated composites are shown in Figure S2 and Table S1 in the Supplementary Materials [43,44,45,46,47,48,49,50,51]. Therefore, it was experimentally and theoretically confirmed that the interconnected filler networks between GNP clusters generated by the segregated structures contributed to the improved electrical conductivities.

**Figure 5:**
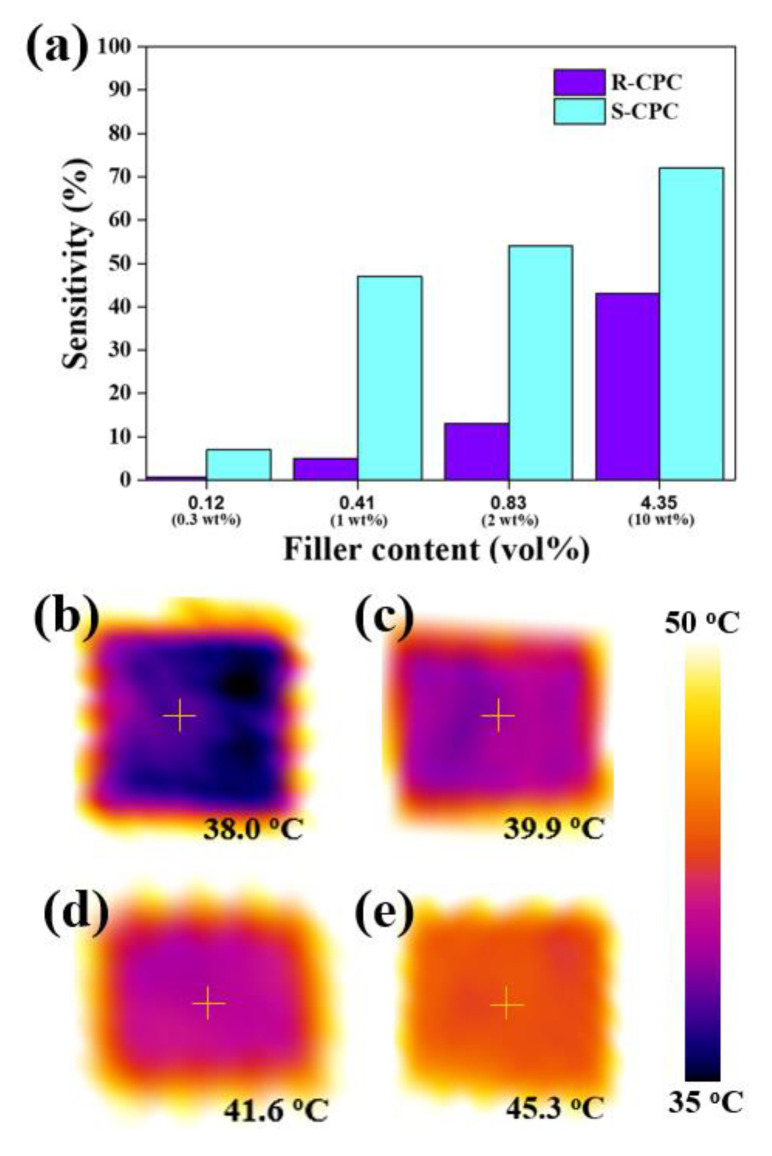
(**a**) Humidity-sensing sensitivity results of the fabricated composites and thermal images of the (**b**) R-CPC2, (**c**) S-CPC2, (**d**) R-CPC10, and (**e**) S-CPC10.

The thermal conductivities of the prepared specimens are displayed in Figure 4b. The thermal conductivity of R-CPC increased linearly with increasing filler content, and the thermal conductivity of R-CPC10 (2.13 W/m∙K) was improved by 610% compared to that of raw PP (0.30 W/m∙K). These results were similar to the thermal conductivity trends of the R-CPCs prepared using the uniform filler dispersion method [52]. Thus, the theoretical thermal conductivity evaluated via Nan’s model based on ITR was in good agreement with the thermal conductivity of R-CPC. In contrast, the thermal conductivity of the segregated composite enhanced linearly to 2 wt% GNP (S-CPC2, 0.69 W/m∙K), then rapidly improved up to 10 wt% (S-CPC10, 4.05 W/m∙K). The thermal conductivity of S-CPCs was evaluated via the percolation model because the experimental thermal conductivity of S-CPC at higher than 3 wt% exceeded that theoretically calculated with Nan’s model. Thermal percolation is a behavior in which the thermal conductivity rapidly improves with increasing contact between the thermally conductive particles [53]. The thermal conductivity of S-CPC evaluated via a thermal percolation threshold ($\emptyset_{tc}$) of 0.45 vol% was in good agreement with the measured value, indicating that the phonon transfer system dominated by the ITR between the PP and GNP in the S-CPC was converted to a system based on direct contact between the GNPs. These results were in good agreement with the partially disconnected network between GNP clusters that contributed to the formation of multiple interfaces (≈ITR) and the fully connected filler networks induced by interconnected GNP clusters, as discussed in Figure 2 and Figure 3, respectively. The observed GNP clusters within the segregated structure of the composite formed by the selective localization of fillers during the process enhanced the electrical and thermal conductivities by inducing effective electron tunneling and phonon transfer. In particular, the fully connected filler network induced the excellent thermal conductivity of the S-CPC by reducing the contribution of ITR. Therefore, it was experimentally and theoretically confirmed that the applied strategy was advantageous for the dramatic improvements in the electrical and thermal conductivities of the composites. In addition, as shown in the humidity sensing sensitivities and thermal images of the fabricated composites (Figure 5), it was confirmed that the sensitivity of the humidity sensor and the heat dissipation performance as a TIM material were improved by the enhanced electrical and thermal conductivity of S-CPC.

## 4. Conclusions

Introducing a segregated network inside the composite is a useful method for achieving excellent conductivities at low contents of conductive fillers. In this study, theoretical and experimental investigations were conducted to discover the improvements in the electrical and thermal conductivities of composites according to the selective localization of GNP filler using the segregated structure and the generation of the conductive network. In the internal structures of the composites observed using FE-SEM and μ-CT, the GNP clusters located at the interfaces of the PP and the fully connected GNP networks were obviously observed, indicating that the applied process was suitable for the fabrication of segregated composites. S-CPC achieved an improved electrical conductivity of 20.79 S/m at a low filler content (1 wt%) compared to R-CPC by achieving the electron tunnel effect generated by GNP clusters on the interfaces of the PP particles. The enhancement trend of thermal conductivity in S-CPC, before the incorporation of filler of 3 wt%, was calculated via Nan’s equation considering ITR; however, the dramatic increase in the measured thermal conductivity at high filler contents (>3 wt%) was evaluated via the thermal percolation equation. This thermal behavior was determined by the formation of a filler network using interconnected GNP clusters, and the enhanced thermal conductivity of S-CPC10 (4.05 W/m∙K) due to a fully connected filler network in the segregated structure was observed. It was confirmed from the experimentally and theoretically evaluated electrical and thermal conductivities of the composites that the segregated structure induced using the applied process was useful for effective electron tunneling and phonon transfer, providing potential options for achieving excellent sensing property and TIM in composites with low filler content.

## Figures and Tables

**Table 1:** Composition of the fabricated composites.

Sample Code	PP, wt% (vol%)	GNP, wt% (vol%)
R-CPC0	100 (100)	0 (0)
S-CPC0		
R-CPC0.1	99.9 (99.96)	0.1 (0.04)
S-CPC0.1		
R-CPC0.3	99.7 (99.88)	0.3 (0.12)
S-CPC0.3		
R-CPC0.5	99.5 (99.80)	0.5 (0.20)
S-CPC0.5		
R-CPC1	99 (99.59)	1 (0.41)
S-CPC1		
R-CPC2	98 (99.17)	2 (0.83)
S-CPC2		
R-CPC3	97 (98.75)	3 (1.25)
S-CPC3		
R-CPC5	95 (97.89)	5 (2.11)
S-CPC5		
R-CPC7	93 (97.01)	7 (2.99)
S-CPC7		
R-CPC10	90 (95.65)	10 (4.35)
S-CPC10		

## Data Availability

Not applicable.

## Supplementary Materials

The following supporting information can be downloaded at: https://www.mdpi.com/article/10.3390/ma16155329/s1, Figure S1: Schematic for fabrication process of R-CPC; Figure S2: Comparisons of percolation threshold ($\phi_{ec}$) and maximum electrical conductivity of segregated composites incorporating GNP; Table S1: Comparisons of materials, fabrication methods and percolation thresholds of segregated composites.
