# Supplementary material for: Enhanced Electrical and Thermal Conductivities of Polymer Composites with a Segregated Network of Graphene Nanoplatelets
Source: Materials (Basel). 2023 Jul 29;16(15):5329. doi: 10.3390/ma16155329 (PMC10420153; doi:10.3390/ma16155329)
Supplement: Supplementary file 1 [file materials-16-05329-s001.zip › materials-2493060-supplementary.pdf]

materials

Supplementary Materials

Enhanced Electrical and Thermal Conductivities of Polymer Composites with a Segregated Network of Graphene Nanoplatelets

Ki Hoon Kim [1,†], Ji-Un Jang [2,†], Gyun Young Yoo [3], Seong Hun Kim [2], Myung Jun Oh [1,*] and Seong Yun Kim [3,*]

1 Department of Carbon Composites Convergence Materials Engineering, Jeonbuk National University, 567 Baekje-daero, Deokjin-gu, Jeonju-si 54896, Jeonbuk, Republic of Korea; kihoon2376@jbnu.ac.kr
2 Research Institute of Industrial Science, Hanyang University, 222 Wangsimni-ro, Haengdang-dong, Seongdong-gu, Seoul 04763, Republic of Korea; jju204@hanyang.ac.kr (J.-U.J.); kimsh@hanyang.ac.kr (S.H.K.)
3 Department of Organic Materials and Textile Engineering, Jeonbuk National University, 567 Baekje-daero, Deokjin-gu, Jeonju-si 54896, Jeonbuk, Republic of Korea; ky5932@gmail.com
* Correspondence: mjoh@jbnu.ac.kr (M.J.O.); sykim82@jbnu.ac.kr (S.Y.K.); Tel.: +82-63-270-2387 (M.J.O.); +82-63-270-2336 (S.Y.K.)
† These authors contributed equally to this work.

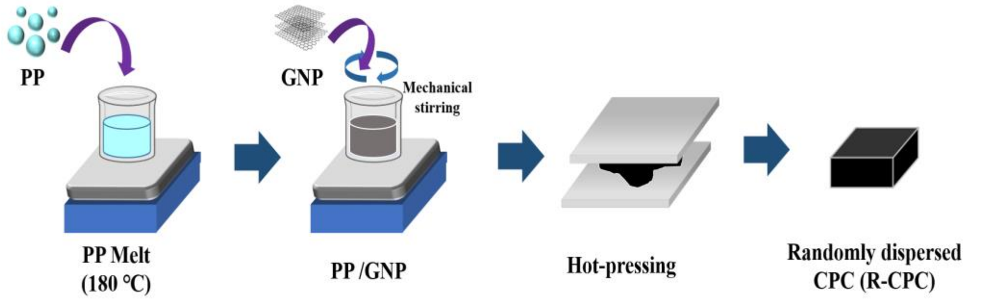

Figure S1. Schematic for fabrication process of R-CPC.

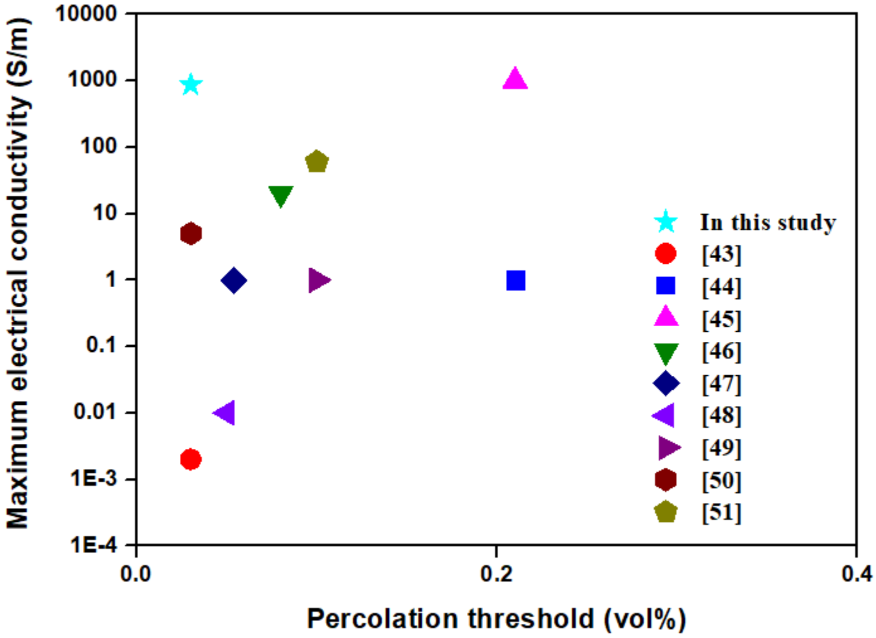

Figure S2. Comparisons of percolation threshold (ϕ_{ec}) and maximum electrical conductivity of segregated composites incorporating GNP [43–51].

Table S1. Comparisons of materials, fabrication methods and percolation thresholds of segregated composites

Matrix§	Filler§	Fabrication method	ϕ_{ec}(vol%)	σ_{max} (S/m)	Reference
PP	GNP	Mechanical mixing	0.04	8.7E+2@6.7vol%	This study
PP	GNS	Latex technology	0.03	2E-3@0.2vol%	[43]
PE	GNP	Mechanical mixing	0.21	1.0E+0@2.6vol%	[44]
PS	GNS	Latex technology	0.21	1.0E+3@4.8vol%	[45]
PS	GNS	Latex technology	0.08	2.0E+1@4.0vol%	[46]
PS	GNP	Electrostatic assembly	0.054	4.6E+1@1.5vol%	[47]
UHMWPE	GNS	Mechanical mixing	0.05	1.0E-2@1.5vol%	[48]
UHMWPE	GNS	Mechanical mixing	0.1	1.0E+0@3.0vol%	[49]
UHMWPE	GNP	Solution mixing	0.03	5.0E+0@0.4vol%	[50]
PASS	GNP	Partial dissolution	0.1	6.0E+1@5wt%	[51]

§PP (polypropylene), PE (polyethylene), PS (polystyrene), UHMWPE (ultrahigh-molecular-weight polyethylene), PASS (poly(arylene sulfide sulfone)), GNP (graphene nanoplatelet), GNS (graphene nanosheet)

References

43. Wang, D.; Zhang, X.; Zha, J.-W.; Zhao, J.; Dang, Z.-M.; Hu, G.-H. Dielectric properties of reduced graphene oxide/polypropylene composites with ultralow percolation threshold. *Polymer* **2013**, 54,1916–1922.
44. Mamunya, Y.; Matzui, L.; Vovchenko, L.; Maruzhenko, O.; Oliynyk, V.; Pusz, S.; Kumanek, B.; Szeluga, U. Influence of conductive nano- and microfiller distribution on electrical conducitivy and EMI shielding properties of polymer/carbon composites. *Compos. Sci. Technol.* **2019**, 170, 51–59.
45. Wu, C.; Huang, X.; Wang, G.; Lv, L.; Chen, G.; Li, G.; Jiang, P. Highly Conductive nanocomposites with three-dimensional compactly interconnected graphene networks via a self assembly process. *Adv. Funct. Mater.* **2012**, 28, 506–513.
46. Pang, H.; Yan, D.-X.; Bao, Y.; Chen, J.-B.; Chen, C.; Li, Z.-M. Super-tough conducting carbon nanotube/ultrahigh-molecular-weight polyethylene composites with segregated and double-percolated structure. *J. Mater. Chem.* **2012**, 22, 23568–23575.
47. Tu, Z.; Wang, J.; Yu, C.; Xiao, H.; Jiang, T.; Yang, Y.; Shi, D.; Mai, Y.-W, Li, R.K.Y. A facile approach for preparation of polystyrene/graphene nanocomposites with ultra-low percolation threshold through an electrostatic assembly process. *Compos. Sci. Technol.* **2016**, 134, 49–56.
48. Pang, H.; Bao, Y.; Lei, J.; Tang, J.-H.; Ji, X.; Zhang, W.-Q.; Chen, C. Segregated conductive ultrahigh-molecular-weight polyethylene composites containing high-density polyethylene as carrier polymer of graphene nanosheets. *Polym. Plast. Technol. Eng.* **2012**, 51, 1483–1486.
49. Wang, B.; Li, H.; Li, L.; Chen, P.; Wang, Z.; Gu, Q. Electrostatic adsorption method for preparing electrically conducting ultrahigh molecular weight polyethylene/graphene nanosheets composites with a segregated network. *Compos. Sci. Technol.* **2013**, 89, 180–185.
50. Hu, H.; Zhang, G.; Xiao, L.; Wang, H.; Zhang, Q.; Zhao, Z. Preparation and electrical conductivity of graphene/ultrahigh molecular weight polyethylene composites with a segregated structure. *Carbon* **2012**, 50, 4596–4599.
51. Yang, J.-C.; Wang, X.-J.; Zhang, G.; Wei, Z.-M.; Long, S.-R.; Yang, J. Segregated poly(arylene sulfide sulfone)/graphene nanoplatelet composites for electromagnetic interference shielding prepared by the partial dissolution method. *RSC Adv.* **2020**, 10, 20817–20826.
